# Educational strategy to develop nursing students’ management competencies in hospital practice

**DOI:** 10.1590/0034-7167-2021-0928

**Published:** 2022-06-24

**Authors:** Laura Andrian Leal, Aline Teixeira Silva, Daniela Sarreta Ignácio, Mirelle Inácio Soares, Nilva Maria Ribeiro, Silvia Helena Henriques

**Affiliations:** I Universidade de São Paulo. Ribeirão Preto, São Paulo, Brazil.; II Centro Universitário de Lavras. Lavras, Minas Gerais, Brazil.

**Keywords:** Professional Competence, Students, Nursing, Education, Higher, Strategies, Hospitals, Competencia Profesional, Estudiantes de Enfermería, Enseñanza Superior, Estrategias, Hospital, Competência Profissional, Estudantes de Enfermagem, Ensino Superior, Estratégias, Hospital

## Abstract

**Objectives:**

to apply and analyze an educational strategy to develop management skills in nursing students to work in hospital practice.

**Methods:**

exploratory, intervention, qualitative study conducted from February 2020 to 2021. Fifty-four nursing students from a public higher education institution participated in this study, in which thirteen workshops were held to discuss management cases. Subsequently, semi-structured interviews were conducted using inductive thematic analysis.

**Results:**

the case studies addressed the management competencies of communication, decision making, leadership, and interpersonal relationships. After the intervention, the strategy was evaluated through interviews, identifying positive aspects regarding knowledge acquisition; and other limiting aspects, such as limited time to discuss the cases.

**Final Considerations:**

the workshops proved to be effective as teaching strategies for students, adding new management knowledge that should help their performance as future nurses, capable of reflection, and the subjects of knowledge construction for the professional practice of nursing.

## RESUMO

**Objetivos:**

aplicar e analisar estratégia educativa para desenvolver competências gerenciais em discentes de Enfermagem para atuação hospitalar.

**Métodos:**

estudo exploratório, de intervenção, com abordagem qualitativa, realizado de fevereiro de 2020 a 2021. Participaram 54 discentes de Enfermagem de uma instituição de ensino superior pública. Aplicaram-se 13 oficinas de discussão com discentes sobre casos gerenciais. Posteriormente, realizaram-se entrevistas semiestruturadas. Utilizou-se análise temática indutiva.

**Resultados:**

os estudos de caso abordaram as competências gerenciais de comunicação, tomada de decisão, liderança e relacionamento interpessoal. Após intervenção, realizou-se mediante entrevistas avaliação da estratégia utilizada, em que foram identificados aspectos positivos quanto a aquisição de conhecimentos; e outros limitantes, como tempo limitado para discussão dos casos.

**Considerações Finais:**

as oficinas mostraram-se eficazes como estratégias de ensino aos discentes, agregando novos saberes gerenciais que deverão auxiliar seu desempenho como futuros enfermeiros, capazes de reflexão, enquanto sujeito construtor do conhecimento para a prática profissional da enfermagem.

## RESUMEN

**Objetivos:**

aplicar y analizar estrategia educativa para desarrollar competencias administrativas en discentes de Enfermería para actuación hospitalaria.

**Métodos:**

estudio exploratorio, de intervención, con abordaje cualitativo, realizado de febrero de 2020 a 2021. Participaron 54 discentes de Enfermería de una institución de enseñanza superior pública. Aplicados 13 talleres de discusión con discentes sobre casos administrativos. Posteriormente, realizadas entrevistas semiestructuradas. Utilizado análisis temático inductivo.

**Resultados:**

los estudios de caso abordaron competencias administrativas de comunicación, toma de decisión, liderazgo y relación interpersonal. Pos intervención, realizada mediante entrevistas evaluación de la estrategia utilizada, en que fueron identificados aspectos positivos cuanto a adquisición de conocimientos; y otros limitantes, como tiempo limitado para discusión de los casos.

**Consideraciones Finales:**

los talleres mostrados eficaces como estrategias de enseñanza a los discentes, agregando nuevos saberes administrativos que deberán auxiliar su desempeño como futuros enfermeros, capaces de reflexión, mientras sujeto constructor del conocimiento para la práctica profesional de enfermería.

## INTRODUCTION

Scientific evidence has characterized the hospital as a place of care that requires trained nurses to deal with various situations. In this context, work overload and the deficit of professional management competencies are associated with factors that negatively affect users’ recovery outcomes^(1-2)^.

The nurses’ competencies can be understood as a sum of their knowledge, skills, and attitudes to perform their functions effectively; this set integrates several elements, including the professional’s knowledge, techniques, behaviors, thinking capacity, and values^(3)^. In this regard, it is observed that the literature has already identified management competencies for the hospital nurse^(1)^, such as interpersonal relationships, materials management, and others, besides those already presented by the National Curricular Guidelines (NCG), namely: communication, decision making, leadership, among others^(4)^. However, there are discussions in which it is questioned whether, in fact, the student can learn and develop those competencies during the undergraduate course^(5)^.

In this sense, we consider that the political and pedagogical project (PPP) of training centers will guide the teaching of undergraduate students and indicate possible learning methodologies by indicating the competencies that need developing during academic training. In this respect, other researchers have shown that management competencies are being addressed in the health area curricula in their PPPs; however, most courses do not have specific disciplines for their development^(6)^.

Thus, institutional strategies for education and improvement in academic training should be a priority and can be developed through changes in undergraduate curricula, including alternative and innovative teaching methods, simulations, and case discussion workshops, providing criticality in the teaching process^(1)^.

About teaching in nursing, in line with these findings, researchers have also evidenced the use of collective didactic-pedagogical experiences, such as problematization, theory-practice integration, role-playing, dramatization, simulation, and group dynamics^(7-8)^.

Moreover, other authors point out educators’ strategies for developing management competencies based on case studies, portfolios, and problem situations in the classroom. These tools are positioned to lead the student to retrieve the knowledge acquired during training and condense it into the construction of knowledge better articulated at the core of management and direct care^(7)^.

The literature shows learning methods based on simulations and case discussions with graduated professionals in specific areas, such as cardiology and ICU. However, there is still little discussion in studies about innovative teaching strategies to develop management competencies in undergraduate nursing students.

Thus, considering the management role of the hospital nurse, the teaching in the academic formation must foster and improve management competencies. For this, it is necessary to use specific strategies that allow the development of/discussion about certain competencies, mainly leadership, decision making, communication, and interpersonal relationship - critical pieces for the actual work process of the hospital nurse^(3)^.

Given these considerations, based on the competencies mentioned, this study presents the following questions: Can group workshops for case discussions assist the teaching-learning of nursing students in improving leadership competencies, decisionmaking, communication, and interpersonal relationships? How do students identify and perceive those teaching strategies?

It is believed that collective learning strategies applied to develop management competencies in nursing students should instigate a professional with innovative and creative performance, capable of social and critical reflection/action as a builder of knowledge for professional nursing practice.

## OBJECTIVES

To apply and analyze an educational strategy to develop management competencies in nursing students to work in hospitals.

## METHODS

### Ethical aspects

This study was developed in accordance with Resolution 466/12 and approved by the Research Ethics Committee (CEP) from the Proponent Institution, letter Nº 0280/2019. The participants signed the Informed Consent Form, and, in order to preserve their anonymity, it was used a coding formed by the letter “S” for “student,” followed by a cardinal numeral indicating the order of the interviews (S1, S2…).

### Design of study

We conducted an exploratory, interventional study with a qualitative approach to data. For the description, we used the Consolidated Criteria for Reporting Qualitative Research (COREQ) instrument, composed of 32 items allocated to three domains: 1) Research team and reflexivity; 2) Study concept, and 3) Analysis and results^(9)^.

### Place and period of study

The study setting was a public Brazilian higher education institution (HEI) in the interior of São Paulo state, and the research took place between February 2020 and March 2021.

### Participants and selection criteria

The study participants were students in the penultimate and last periods of two courses at the selected HEI: bachelor’s degree in Nursing; and bachelor’s and BSc degree in Nursing. As selection criteria, students from the last periods of the undergraduate course, taking the discipline “Organization and management in hospital nursing,” were invited, a period in which they could put the content learned into practice. The total number of HEI students was 110. The reason for choosing students from the last periods was because, at that moment, they already had a learning experience and understood most of the competencies necessary for the work of the hospital nurse.

### Collection and organization of data

All students in the sample were formally and personally invited through an invitation letter (outside of class time) and electronically to participate in the research activity to be carried out, with the day, time, and place scheduled. After accepting the ethical term, management case discussion workshops were implemented to develop these competencies. It is highlighted that this activity was carried out in two ways: on-site (in the teaching institution) and online (in Google Meet rooms) due to the current context of the Coronavirus Disease 19 (COVID-19) pandemic. All sessions were recorded.

Thus, the face-to-face intervention workshops were held in the HEI in large classrooms with round tables to facilitate the discussion process, providing a cozy and private environment. The online intervention workshops were carried out through a Google Meet room, in which the participants opened the microphone and camera to meet the process of in-depth and effective discussion. Both modalities of reunion (on-site or online) occurred outside of class time, according to the availability of students, but it was difficult to schedule all of them.

The methodological reference used for the development of the workshops was the Marquis-Huston Critical Thinking Teaching Model^(10)^, based on the four overlapping spheres. During the workshops, each sphere was practiced: didactic theory, problemsolving, group process, and personalized learning.

To carry out this activity, first, a self-administered, easy-to-fill-out, sociodemographic data questionnaire was made available in person and/or electronically (through Google Forms); and a brief PowerPoint explanation was used about nurses’ management competencies. Next, the students were offered a formal approach, presenting them with strategies for working in groups. Two case studies were developed (presented below) that encouraged nurses’ learning of management competencies in the hospital context.

Case 1: You arrived at work at a private hospital in the interior of São Paulo for the night shift in the medical clinic sector and were informed by the nurse on duty in the afternoon that Ward A of your sector had a reduced number of nursing staff due to sick leave. In addition, the nurse on Ward B in this same sector was absent without informing her manager, and this is a recurring problem. As your colleague said, you had to take over both Wards. It is important to emphasize that communication problems can generate unsafe acts that affect everyone on the team and can even affect the quality of care provided. Based on this situation, you redistributed the activities of the two nursing teams. However, each worker was left with many nursing activities. The employee assigned to the medication was newly admitted to the sector and, while performing her tasks, she became very stressed with this situation, so she asked for your help to clarify the preparation of a time-scheduled medication; and you, in the hustle and bustle of the sector, ended up not giving her that much attention. Then, the professional confused the medication labels, which generated an adverse event, putting the patient’s life at risk. The professional informed you of what happened, and measures were taken regarding the employee, the staff, and the patient. After the patient’s condition stabilized, you gathered the whole team and explained what happened, preserving the employee’s identity. At the end of the shift, you filled out a document and delivered it to your boss, reporting what happened and asking for a decision.

Case 2: Because of the exaggerated increase in costs, the administrative management of a private hospital in the interior of São Paulo decided to reduce the number of nursing assistants and technicians, which has generated an increase in work demand, including for the nurses themselves. Dismissals occurred without explanations. There was no prior communication from the management about this decision. Due to this, the number of employees for each shift has been reduced by 20%. This situation generated a tense atmosphere in the sector among the nursing team, leading to conflicts or stressful situations in the relationship, which can significantly harm patient care. In this regard, you witnessed two nursing technicians arguing in the central corridor and had to take action. In addition, the administration reiterates the need to manage material resources in the care units. Nursing represents the largest contingent of professionals in any sector, so it uses many material resources to perform care activities. The nurse was aware of this and needed to talk to the team. In addition, the nurse has constantly observed excessive and inadequate use of sterile gloves in clean, non-invasive procedures. Since the nurse has already been warned that financial resources have been reduced, sound decisions must be made with the staff.

The cases were validated and previously tested by specialists, masters, and doctors in Nursing Management experts in pedagogical strategies. The instruments for validation were made available by e-mail to the reviewers, who had 15 days to make recommendations.

The types of case studies were addressed randomly among the meetings. According to their availability, there were 13 case discussion workshops, with an average of seven participants each, with an average duration of 45 minutes.

Furthermore, 60 days after the case discussion workshops and after the immersion of students in the internship field, interviews were conducted with the students who participated in the selected activity. They were carried out individually, on-site, and online to analyze the undergraduates’ experience with the intervention and other learning methods available to develop management competencies. The interviews had an open-ended question guide, tested, and validated by experts, and were conducted according to the participants’ availability (date, time, and place). They were recorded and lasted approximately 20 minutes.

### Data analysis

The discussions originated from the workshops, as well as the interviews were transcribed and subjected to inductive thematic analysis for data interpretation, going through the following steps: transcription and reading of the data; systematic coding of interesting data features; search for topics by grouping codes; review and verification of the topics to which they respond for extraction of coding; analysis to refine the details of each topic; and final analysis of selected excerpts concerning the research guiding questions^(11)^.

## RESULTS

A total of 110 students were formally invited. Fifty-four (49%) participated in the intervention stage through 13 workshops, of which 43 (79.62%) took part in the second stage of the study, that is, the interviews. About refusal, 50 (45.45%) students refused due to personal commitments, and another 6 (5.45%) refused for no apparent reason.

Of the participants, 47 (87%) were female, and the age ranged from 20 to 52 years, with a mean of 23.93 years. They were from different states, such as São Paulo, Minas Gerais, Ceará and Rio de Janeiro. Regarding the course modalities, 31 (57.4%) were students of the bachelor’s degree in Nursing, and 23 (42.59%) of the bachelor’s and BSc Degree in Nursing.

Regarding the intervention stage, five (38.46%) workshops were on-site, held from February to March 2020; and eight (61.53%) were online, from April to December 2020.

Both management cases presented in the workshops were intended to foster discussion and interventions for the development of specific management competences; and to make this possible, there were guiding questions that enhanced the problematization of the competences involved: What are the problems of the case reported? List the management competencies that should be used according to this problematization. How can nurses solve the problem of lack of communication? How do you think conflicts should be dealt with? What solutions will the nurse propose to his/her team? How can the nurse reduce inadequate spending on materials?

Case 1 was proposed to enhance communication, decision-making, and leadership competencies, and the second case was to develop management competencies in communication, decision-making, and interpersonal relationships.

During the workshops, the students could speak freely about the situations presented, using their previous knowledge; and the questions were only used if the stimulus for discussion was needed and to favor the development of the proposed competencies.

Thus, during the workshop discussions, the students expressed several problems, identifying nurses’ competencies and possible solutions, presented below.

For the problems raised for Case 1, the students highlighted communication failure, reduced number of employees, fragmentation of care, a new employee without training, and work overload. For such problems, they listed several possibilities for resolution. First, talk to the nurse to understand the reasons for her absence and give her a warning for it. Communicate with the superior using written communication and technology (WhatsApp). Redistribute activities according to performance, adequate dimensioning, and similar materials/medications organization. Communicate with the physician explaining the medication error, observe the patient, and talk in a non-punitive way with the technician who made the error, using/formulating the unit’s protocols for error. Supervise activities of the newly hired team, offer shared decisions to solve the unit’s problems, using simulation to train the team. Thus, the competencies were problematized and developed while discussing the possibilities of solving the case.

Moreover, the discussions permeated the development of competencies already intended and fostered the development of others, such as interpersonal relationships and continuing education.

In Case 2, were identified problems such as dismissals without prior communication, reduced number of employees, tense work environment due to the dismissals, work overload, conflict in the corridor where patients/companions circulate, and inappropriate use of materials. They highlighted the nurse’s possible forms of resolution: meet with the superior stressing the size and impacts of work overload, communicate the team about the decrease of employees, redistribute activities according to performance and preference, meet with each technician individually listening to him/her carefully, provide continuing education on sterile and non-sterile materials, supervise the team, register information and materials for cost, and give each professional of the team a feedback on the positive points.

In this way, through discussions, the students proposed the resolution of problems improving the development of competencies such as communication, interpersonal relationships, decision making, continuing education, supervision, and leadership.

Thus, the cases problematized in the workshops promote the discussion and improvement of the competencies necessary to face them. It is noteworthy that, in all workshops, several competencies were problematized since nurses integrate several of them in their work process.

Two months after the intervention through workshops, the researcher contacted the participating students to evaluate the effectiveness of the intervention through individual interviews. It is worth noting that, at that point, all students had already completed the management internship and were already immersed in the discipline Supervised Curricular Internship Subject in the last period before graduation.

In the interviews, the students could indicate positive and limiting points of the intervention strategy. They identified positive aspects of the intervention with significant consequences for learning. We highlight its contribution to the immersion in practice; complementation of knowledge gaps on the content of management skills offered by the discipline of hospital management; ways to deal with the management of people and material resources in nursing practice; full training for the labor market; and means to deal with conflicting situations:

*The workshops gave support that was missing from the disciplines. It was good because it was in a group, we exchanged experiences, I could see how to work with material and people management. I could also prepare myself to deal with conflicts.* (S29)

*It was extremely positive for me because it allowed me to learn about leadership, how to behave as a leader, lead, communicate effectively, and solve conflicts and group problems to strengthen the relationship in the team, supervision, and so on. I was able to think and reflect on my posture as a nurse, something never problematized before from the management point of view.* (S19)

*We even used cases and simulations in previous disciplines, but it was something focused on the clinic and pathophysiology. In the internship, I experienced all this, which helped; having done the discussion and then going to the supervised practice was essential. In addition, it also helped me in my residency, and the proficiency exam because I knew about management issues.* (S40)

As for limiting aspects, the statements showed that the limitation was more related to the dynamics of the strategy and not to the content developed, like reduced time for discussing the cases. They also highlighted limitations in implementing learning during the immersion in the practice scenario, revealing the effectiveness of the developed strategy, according to the statement:

*As a limiting aspect, I think there was little time to discuss the cases because I participated in two, and one had too many people. You cannot cover all the management competencies in a single study. Besides, the internship hospital’s policy and some supervisors did not grant much autonomy to practice the lessons learned in the workshop.* (S12)

*I stayed in a place where the internship team was not receptive and would not let me do anything, so I could not put into practice the lessons we learned.* (S7)

Furthermore, the interviews also included questions related to other learning strategies that the students experienced and considered significant for the development of management competencies, including the use of assessment tests before and after learning, simulations, practical laboratories, group discussions, classes with video resources, and the internship in the field with the complete mediation from the nurse supervisor:

*We experience teaching-learning strategies, such as simulation, and it is a very enriching strategy to consolidate theory and gain experience. In the resolution of cases, we did pre and post-tests before theory, labs, videos, and internships where the nurse helps and gives us autonomy, which also helps a lot.* (S24)

*We could have had a simulation in decision-making management, you know? I think this could also have helped us to be more competent.* (S49)

## DISCUSSION

The current challenges imposed on education have led to searching for strategies that enhance the teaching-learning process. It is believed the traditional educational proposal, centered on the professor, who teaches the subject through expository lectures, is inappropriate for fostering discussions and critical reflections on a given topic.

Therefore, to implement innovative teaching strategies with undergraduate nursing students, this investigation focused on conducting an intervention utilizing case study workshops to develop and analyze the learning of nurses’ management competencies to work in hospitals. It is well known that the case study discussion method allows interdisciplinarity, being possible to relate and use knowledge among undergraduate disciplines to place a concept within a specific context. In nursing, that tool provides the possibility of connecting knowledge among the various disciplines of the undergraduate course^(12)^, including, in this process, managerial content, especially the management competencies of communication, interpersonal relationships, leadership, and decision making.

As for implementing that, leadership teaching is seen as a challenge since it should be subsidized not only by the theoretical approach but also by the practical exercise, as done in the intervention guided by the case study. Thus, we notice the importance that leadership be inserted as a transversal axis in undergraduate courses and not as an isolated topic. Consequently, the formation of nurse leaders depends on an education that promotes the teaching of leadership obliquely in its curriculum, aiming to develop a critical and reflective approach to reality with beneficial changes to improve the current scenario^(13-14)^.

However, reviews of the literature at the international level consider leadership as a way to influence, support, and motivate teams to achieve goals and obtain recognition through rewards. Moreover, the leader is a person with clear communication, persuasion, strategic planning, negotiation, and conflict resolution skills to ensure the quality of care^(15)^, an aspect also addressed by the students.

Hence, when fostering discussions during the implementation of leadership development, the groups of students also established the importance of the leader using shared management, highlighting actions such as listening to their collaborators and providing opportunities for joint decision-making. In this sense, shared leadership incorporates the notion of mutual reciprocity, as opposed to the idea of the unidirectional influence of a leader over his/hers followers; instead, he/she acts as a facilitator^(16)^. In the case of nursing, the leader’s figure implies guiding his team so that everyone participates in the decision-making processes.

Therefore, the implementation of leadership competence development led to the discussion about the importance of nursing supervision. When using shared management, the nurse leader must provide oversight to avoid errors or even cost containment, as explained in the cases presented. The control and handling of materials are part of the nurse’s attributions. However, this process can be collaborative with other nursing team members, and supervision by the sector nurse is indispensable, an aspect much discussed in the workshops.

Furthermore, it is observed that the leader, when leading, influencing, and supervising a team, makes managerial decisions, and this process is interconnected; thus, managerial decision making was another competence worked on with the nursing students in the workshops. Following this line of thought, the act of deciding is a complex cognitive process, defined as the choice of a particular course of action. Problem-solving is part of the decision process and represents a systematic way that focuses on analyzing a problematic situation. A problem usually can trigger a decision-making process, although that could happen without concentrating on the real cause of the conflict or doing nothing about it^(10)^.

However, it was observed that the students had more difficulty in problematizing this competence in the proposed workshops. That is possibly due to inexperience, lack of reflection and encouragement about the decision-making processes that are the responsibility of nurses in their daily work by the professors or internship supervisors, or even because the course hours are not sufficient to contemplate the complete learning of this competence.

In this regard, case studies with real situations help develop competencies, decision-making, and problem-solving in professional practice. Moreover, it is emphasized that simulation may be an ideal method to develop decision-making skills, not only for the education of nurses but also to evaluate the competencies of advanced nursing practice in the management category^(17)^.

However, it is worth noting that the competencies cannot be seen in isolation but interacting and/or influencing each other. Thus, only when the team members’ relationship is respected will the competencies mentioned above wholly develop. In this perspective, the interpersonal relationship was another competence worked on with the students through reflection on managing conflicts and establishing a harmonious and stable connection. In this regard, the literature brings the concept of relational competence as the ability to form and maintain relationships, regardless of the type, among close peers^(18)^.

About this premise, in the view of the students, interpersonal relationship competence was attributed mainly to the category of conflict resolution, using shared leadership, clear communication, calm, and politeness. Therefore, because the nursing student does not have practical experience, it is typical for him/her to have insecurities and a lack of ability to deal with conflict situations and relationships within their work team. Consequently, it is essential to align the preceptors and professors in articulating all the content offered in the course to substantiate and guide the practice during the internship^(19)^.

Given this, one way to ensure a harmonious environment and, consequently, a good relationship is through professional acknowledgment and appreciation. The students considered that bonus and appreciation schemes could be excellent methods to ensure good connections in the field. Some studies also suggested that different bonus payment schemes can influence job quality^(20-21)^.

It is worth mentioning that several competencies are required from the nurse manager. Thus, institutions demand ethical posture and attitude from this professional to achieve goals and excellence in teamwork; to accomplish that, interpersonal communication is essential. In this sense, students considered communication competence an indispensable process for patient safety, conflict managing, and the progress of the entire shift. Communication as a basic competence can happen in several ways: verbal, written, and personal. However, those forms may be insufficient when used exclusively, compromising the organization’s objectives^(22)^. In this context, the students problematized possibilities to achieve horizontal communication since it would promote the relationship between users and contributors, besides standardizing actions and sharing knowledge and values.

It is noteworthy to highlight that teaching how to communicate effectively can be challenging due to the variety of potentially tricky conversations that nursing students face in clinical settings^(23)^. For this reason, professors should use a variety of teaching methodologies and real-life case situations to foster communication development^(24)^.

After the group intervention, it was necessary to evaluate the method applied to receive feedback from the participants. The evaluation showed good acceptance of the educational method regarding management competencies, mainly by the joint construction of knowledge. The reports demonstrate content learning by the students, as many reported that they could experience practical situations and obtain positive results in proficiency exams in the nursing management area. Many suggested the implementation and continuity of this teaching-learning method during their academic training, revealing the method’s contributions.

With specific case studies and group discussions, the strategy employed revealed that knowledge accumulates through the exchange of understandings and ideas among all participants.

The use of problematizing methods with joint construction, such as the one used in this study, should fill gaps in the management area less contemplated by the curricula of undergraduate students. That converges with the work of other researchers who have evidenced the benefits of cooperative learning in small groups, judging that communication skills, critical thinking, and advances in teamwork are essential in this process^(25)^.

Finally, the evaluation of the educational intervention also raised discussions about other methods to develop management competencies. During the implementation of the interventions, the students pointed out the pre- and post-learning tests, audiovisual resources such as videos and simulations, and the supervised internship with the total support of the nurse supervisor. These findings corroborate the results present in the international literature, which point in the direction of high-fidelity simulation strategies, combined learning environment, cooperative learning method, and management case study^(24)^.

### Study limitations

It is necessary to develop other scientific investigations that contemplate innovative teaching strategies with undergraduate nursing students, addressing different management competencies required for their academic training.

### Contributions to the field

The elaboration of research focused on implementing innovative teaching strategies for the development of management competencies in Nursing students should ensure quality nursing practice. Moreover, it fills gaps in training and contributes to flexible education systems aimed at training professionals with profiles that meet the demands of the labor market.

## FINAL CONSIDERATIONS

This study results showed the use of a teaching strategy that contributed to improving nursing student education regarding management competencies for the hospital context. The discussion workshops allowed to implement leadership, decision-making, interpersonal relationship, and communication competencies. Thus, they filled possible gaps in the training of undergraduate nursing students concerning knowledge, skills, and attitudes in the management area. That contribution prepares them to act in the future as nurses, with effectiveness in their management work process.

In this sense, professors of higher education institutions must list and implement different strategies and teaching methods as possibilities for the development of management competencies in academic nursing training. That should occur both in the classroom environment and in the context of practice, with the innovation of technologies that can help the future nurse manager. Moreover, it is known that the teaching process involves the efforts and attitudes of educators and students, and therefore, it depends on the professor’s ability to master content and learning techniques necessary to instigate active postures in students that lead to their learning.

## References

[1] Ferracioli GV, Oliveira RR, Souza VS, Teston EF, Varela PLR, Costa MAR (2020). Management competencies in the perspective of nurses in the
hospital context. Enferm Foco [Internet].

[2] Graan ACV, Williams MJS, Koen MP (2016). Professional nurses' understanding of clinical judgement: A
contextual inquiry. Health SA Gesondheid.

[3] Fukada M (2018). Nursing Competency: Definition, Structure and
Development. Yonago Acta Med.

[4] Ministério da Educação (BR) (2001). Resolução CNE/CES nº 3/2001 sobre as Diretrizes Curriculares Nacionais
do Curso de Graduação em Enfermagem[Internet].

[5] Leal LA, Soares MI, Silva BR, Brito LJS, Bernardes A, Henriques SH (2019). Professional competencies for hospital nurses: a documentary
analysis. Rev Enferm Cent O Min.

[6] Trombelli FSO, Ferreira AMD, Oliveira JLC, Marcon SS, Matsuda LM (2018). Management skills, curricular analysis of courses in the public
health area. Rev Saúde Comum [Internet].

[7] Pront L, Mcneill L (2019). Nursing students' perceptions of a clinical learning assessment
activity: 'linking the puzzle pieces of theory to practice'. Nurse Educ Pact.

[8] Barbosa LR, Cavalcante MBG, Pereira LL (2018). Desafios vivenciados por docentes no ensino das competências
gerenciais. Rev Cubana Enferm [Internet].

[9] Souza VRS, Marziale MHP, Silva GTRS (2021). Translation and validation into Brazilian Portuguese and
assessment of the COREQ checklist. Acta Paul Enferm.

[10] Marquis BL, Huston CJ (2015). Administração e liderança em enfermagem: teoria e aplicação.

[11] Braun V, Clarke V (2006). Using thematic analysis in psychology. Qual Es Psychol.

[12] Tsimane TA, Downing C (2020). Transformative learning in nursing education: a concept
analysis. Int J Nurs Sci.

[13] Knop AL, Gama BMBM, Sanhudo NF (2017). Nursing students and leadership development: challenges facing in
the curriculum internship. Rev enferm Cent.-Oeste Min.

[14] Kaso N, Mariani M, Ildam D, Firman F, Aswar N, Iksan M (2021). The Principal's Leadership: How to Improve the Quality of
Teaching and Learning Process in State Junior High School of
Luwu. J Adm Kaso.

[15] Reed BN, Klutts AM, Mattingly TJ (2019). A Systematic Review of Leadership Definitions, Competencies, and
Assessment Methods in Pharmacy Education. Am J Pharm Educ.

[16] Linares PF, Higueras ER, Martin-Ferreres L, Torre MAC, Capellades LW, Fernández-Puebla AG (2020). Dimensions of leadership in undergraduate nursing students:
validation of a tool. Nurs Educ Today.

[17] Bryant K, Aebersold ML, Jeffries PR, Kardong-Edgren S (2020). Innovations in simulation: nursing leaders' exchange of best
practices. Clin Simul Nurs.

[18] Cashen KK, Grotevant HD (2020). Relational Competence in emerging adult adopters: conceptualizing
competence in close relationships. J Adult Dev.

[19] Mamaghani EA, Rahmani A, Hassankhani H, Saunders C, Dean S, Ferguson C (2019). Effective Characteristics of Iranian Nursing Students in Their
Relationship with Clinical Nurses. J Caring Sci.

[20] Beaman L, Keleher N, Magruder J (2018). Do job networks disadvantage women? evidence from a recruitment
experiment in Malawi. J Labor Econ [Internet].

[21] Pieper JR (2015). Uncovering the nuances of referral hiring: how referrer
characteristics affect referral hires’ performance and likelihood of
voluntary turnover. Pers Psychol.

[22] Holly C, Poletick EB (2014). A systematic review on the transfer of information during nurse
transitions in care. J ClinNurs.

[23] Neilson SJ, Reeves A (2019). The use of a theatre workshop in developing effective
communication in pediatric end of life care. Nurse Educ Pract.

[24] Gutiérrez-Puertas L, Márquez-Hernández VV, Gutiérrez-Puertas V, Granados-Gámez G, Aguilera-Manrique G (2020). Educational interventions for nursing students to develop
communication skills with patients: a systematic review. Int J Environ Res Public Health.

[25] Abramczyk A, Jurkowski S (2020). Cooperative learning as an evidence-based teaching strategy: what
teachers know, believe, and how they use it. J Educ Teach.

